# Neuronal Intra-Individual Variability Masks Response Selection Differences between ADHD Subtypes—A Need to Change Perspectives

**DOI:** 10.3389/fnhum.2017.00329

**Published:** 2017-06-28

**Authors:** Annet Bluschke, Witold X. Chmielewski, Moritz Mückschel, Veit Roessner, Christian Beste

**Affiliations:** ^1^Cognitive Neurophysiology, Department of Child and Adolescent Psychiatry, Faculty of Medicine, Technische Universität DresdenDresden, Germany; ^2^Experimental Neurobiology, National Institute of Mental HealthKlecany, Czechia

**Keywords:** ADD, ADHD-C, event-related potentials, residue iteration decomposition, conflict processing

## Abstract

Due to the high intra-individual variability in attention deficit/hyperactivity disorder (ADHD), there may be considerable bias in knowledge about altered neurophysiological processes underlying executive dysfunctions in patients with different ADHD subtypes. When aiming to establish dimensional cognitive-neurophysiological constructs representing symptoms of ADHD as suggested by the initiative for Research Domain Criteria, it is crucial to consider such processes independent of variability. We examined patients with the predominantly inattentive subtype (attention deficit disorder, ADD) and the combined subtype of ADHD (ADHD-C) in a flanker task measuring conflict control. Groups were matched for task performance. Besides using classic event-related potential (ERP) techniques and source localization, neurophysiological data was also analyzed using residue iteration decomposition (RIDE) to statistically account for intra-individual variability and S-LORETA to estimate the sources of the activations. The analysis of classic ERPs related to conflict monitoring revealed no differences between patients with ADD and ADHD-C. When individual variability was accounted for, clear differences became apparent in the RIDE C-cluster (analog to the P3 ERP-component). While patients with ADD distinguished between compatible and incompatible flanker trials early on, patients with ADHD-C seemed to employ more cognitive resources overall. These differences are reflected in inferior parietal areas. The study demonstrates differences in neuronal mechanisms related to response selection processes between ADD and ADHD-C which, according to source localization, arise from the inferior parietal cortex. Importantly, these differences could only be detected when accounting for intra-individual variability. The results imply that it is very likely that differences in neurophysiological processes between ADHD subtypes are underestimated and have not been recognized because intra-individual variability in neurophysiological data has not sufficiently been taken into account.

## Introduction

Attention deficit/hyperactivity disorder (ADHD) is a multi-faceted developmental disorder (Kieling and Rohde, 2012) in which different subtypes can be distinguished based on clinical criteria. The most prominent are the predominantly inattentive subtype (attention deficit disorder, ADD) and the combined ADHD subtype (ADHD-C; Randall et al., 2009; Ahmadi et al., 2014). As stressed by the research domain criteria (RDoC) initiative, there is an increasing need to validate existing clinical classifications and to generate classifications on the basis of neuronal (neurophysiological) data (Insel et al., 2010). In this regard, neuronal correlates of cognitive control and action selection mechanisms are of interest given the prominent executive function deficits in ADHD (Houghton et al., 1999; Kenemans et al., 2005; Roberts et al., 2012). Conflict monitoring abilities play a crucial role in this context (Randall et al., 2009; Cao et al., 2013; Bluschke et al., 2016b, 2017; Stroux et al., 2016) and have been suggested as an endophenotype of ADHD (Albrecht et al., 2008). Concerning subtype differences, patients with ADHD-C have been shown to be somewhat more affected by such executive control deficits (Nikolas and Nigg, 2013; Ahmadi et al., 2014; Bluschke et al., 2016a,c; Dobson-Patterson et al., 2016).

Yet, critically, such executive control processes are affected by a multitude of cognitive subprocesses from perceptual and attentional selection to response selection and motor processes (Barkley, 1997; Houghton et al., 1999; Nikolas and Nigg, 2013). These different subprocesses are important to consider when aiming to establish dimensional cognitive-neurophysiological differences between ADD and ADHD-C. This is due to the fact that a similar behavioral outcome may emerge from dysfunctions at different processing levels. While it is possible to examine the neurophysiology of these different processing stages in ADD and ADHD-C using classic event-related potential (ERP) methods (Johnstone and Clarke, 2009; Gong et al., 2014; Mazaheri et al., 2014), it is important to consider that this and related methods can only yield a true reflection of the neural activity when there is little intra-individual variability (Ouyang et al., 2011, 2015a; Mückschel et al., 2017). This however, is unlikely to be the case in ADHD because a high behavioral intra-individual variability is a core aspect also discussed as an endophenotype of this disorder (Henríquez-Henríquez et al., 2014; Lin et al., 2015; Saville et al., 2015). There may thus be a considerable bias in knowledge about altered neurophysiological processes in patients with ADHD and its different subtypes. When aiming to establish dimensional cognitive-neurophysiological constructs of ADHD (refer to the RDoC initiative; Insel et al., 2010), it is crucial to eliminate this bias and to explore, how dissociable cognitive-neurophysiological subprocesses differ between patients with different ADHD subtypes.

To achieve this, we use residue iteration decomposition (RIDE; Ouyang et al., 2011, 2015a,b) applied on single-trial ERP data in combination with source localization techniques to examine neurophysiological changes at different levels in the information processing stream in ADD and ADHD-C. For this, participants performed a classical flanker task which reliably measures conflict and interference control (Keye et al., 2013; Chmielewski et al., 2014; Bluschke et al., 2016b) and has already been extensively used in ADHD research (e.g., Jonkman et al., 1999; van Meel et al., 2007; Albrecht et al., 2008; Mullane et al., 2009; Iannaccone et al., 2015). However, to the best of our knowledge, no study has so far actually statistically accounted for alterations in intra-individual variability when interpreting results. RIDE offers a way of doing just that. It decomposes EEG data on the basis of their timing and timing variability properties and is therefore well-suited to address issues related to a possible bias caused by intra-individual variability. Using conventional techniques, this aspect of the data cannot be sufficiently accounted for. RIDE calculates three clusters of components locked to the target stimulus of the trial with dissociable functional relevance (Ouyang et al., 2011, 2015a): the S-cluster refers to processes related to the stimulus (like perception and attention), the R-cluster refers to processes related to the response (like motor preparation and execution) and the C-cluster refers to intermediate processes between S and R (like stimulus evaluation and response selection). The different components within a RIDE cluster correspond to the traditional components in the conventional ERP. The correspondence of specific peaks and topographies, however, crucially depends on the proximity to the locking point and the varying degrees of variability in the neurophysiological signal resulting from it (Ouyang et al., 2015a,b). Specifically, RIDE-components in the S-cluster commonly only differ very little from the corresponding ERPs like the P1 and N1, as they both are closely locked (and thus immediately related) to the stimulus. As the proximity to the locking point increases, the intra-individual variance also rises. Based on the removal of this variability by RIDE, there are more differences in latency and scalp topography between the RIDE-component (i.e., the C-cluster) and the corresponding ERP (like the P3; Mückschel et al., 2017). These are larger than it is the case in the S-cluster. Based on the common core attentional deficits, we thus hypothesize that stimulus processing and attentional selection mechanisms do not differ between ADHD-C and ADD. In contrast, processes related to response selection and decision processes are expected to be reduced in the ADHD-C group, as most previous research results suggest for executive control deficits to be more pronounced in this subgroup (Nikolas and Nigg, 2013; Ahmadi et al., 2014; Dobson-Patterson et al., 2016). We further do not expect any motor processing deficits as behaviorally they are not specific to one subtype (Ghanizadeh, 2010). Importantly we hypothesize that due to the known high intra-individual variability, these differential effects will only be found after RIDE-analysis but not while using standard ERP methodology. To specifically focus on dimensional neurophysiological constructs representing symptoms of and differences between ADD and ADHD-C, we deliberately do not focus on the contrast between these groups of patients and healthy controls. Instead, we compare groups of patients with ADD and ADHD-C who do not differ in interference control behavior in the applied task. Controlling for differences on the behavioral level makes it possible to exclude potential confounding effects on neurophysiological data. Despite the complex and not necessarily direct associations between electrophysiological and behavioral measures, such differences may amplify neurophysiological differences between the examined ADHD subtypes.

## Materials and Methods

### Sample

Only patients in whom ADD or ADHD-C diagnoses had been determined according to standard clinical procedures (incl. parent and child interview, teacher report, symptom questionnaires, IQ testing, exclusion of underlying somatic disorders via EEG, ECG, audiometry and vision testing) were included in the study. All participants fulfilled criteria for ADHD according to ICD-10 criteria (F90.0, F90.1 (ADHD-C) or F98.8 (ADD)). All participants were regular patients of the outpatient clinic of the Department of Child and Adolescent Psychiatry, University Hospital Dresden. Patients were excluded if additional severe or acute psychiatric (e.g., autism, tics, depressive episode) or somatic comorbidities had been diagnosed. For the descriptive biographical and clinical data, the mean and the standard deviation are given. Overall, 34 patients with ADHD-C (1 female, 11.0 ± 2.5 years, age range between 7 years and 15 years, IQ: 101.7 ± 13.9, 17 medicated with extended-release Methylphenidate) and 25 patients with ADD (3 female, 10.9 ± 1.9 years, age range between 7 years and 15 years, IQ: 94.0 ± 14.3, 14 medicated with extended-release Methylphenidate) participated in the study. The two groups were recruited simultaneously and did not differ regarding age (*t*_(57)_ = 0.08, *p* = 0.93) or IQ (*t*_(57)_ = −1.8, *p* = 0.08). In the ADHD Symptom Checklist (Döpfner et al., 2008), parents rated (0: no problems, 3: severe problems) their children in regards to inattention (average raw score ADHD-C: 2.2 ± 0.50, ADD: 2.0 ± 0.41, *t*_(57)_ = −2.3, *p* = 0.03), hyperactivity (average raw score ADHD-C: 1.7 ± 0.61, ADD: 0.59 ± 0.47, *t*_(57)_ = −7.8, *p* ≤ 0.001) and impulsivity (average raw score ADHD-C: 2.3 ± 0.39, ADD: 1.1 ± 0.59, (*t*_(57)_ = −9.6; *p* ≤ 0.001), thus confirming ADHD symptomatology and the two different subtypes. All subjects and their parents or legal guardians provided written informed consent in accordance with the Helsinki Declaration of 1975, as revised in 2008. The study was approved by the local ethics committee of the Medical Faculty of the TU Dresden.

### Task

**Figure 1:**
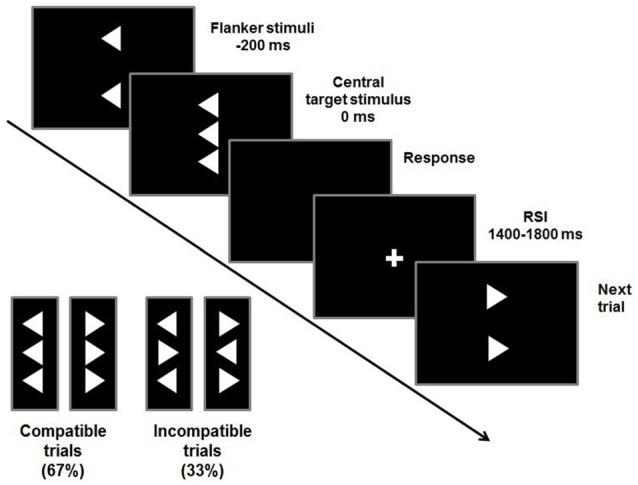
Stimuli and timings of a flanker trial. Compatible or incompatible flanker arrows are presented 200 ms before the target stimulus. Participants are required to indicate the direction of the target stimulus by pressing a button accordingly. The response-stimulus interval (RSI) varies between 1400 ms and 1800 ms.

To examine conflict monitoring processes, a flanker task was used (Beste et al., 2010, 2013). Flanker tasks are traditionally used to examine interference control in ADHD (Mullane et al., 2009). In the Flanker task used, vertically arranged visual stimuli are presented with the target stimulus (white arrowhead) in the center of the screen on a black background (see Figure 1).

Target stimuli pointing to the left or right are flanked by two vertically adjacent arrowheads pointing in the same (compatible) or opposite (incompatible) direction as the target stimulus. These flanker stimuli precede the target by 200 ms. To adapt the paradigm to children, the stimulus onset asynchrony had been prolonged by 100 ms compared to the adult version (Beste et al., 2013; Bluschke et al., 2016b). This maximizes the degree of conflict induced by the flankers and the likelihood of premature responding to the flankers. The target is displayed for 300 ms and flanker stimuli are switched off simultaneously. The response-stimulus interval (RSI) is pseudo-randomized between 1400 ms and 1800 ms. To further increase the level of conflict, time pressure is administered by asking the participants to respond within 450 ms. In case responses exceeded this deadline, an auditory warning stimulus (1000 Hz, 60 dB SPL) was given 1200 ms after the response. This stimulus setup was used in two blocks of 120 stimuli each. Out of these 240 trials, 67% were compatible and 33% were incompatible trials.

### EEG Recording and Analysis

The EEG was recorded from 60 Ag/AgCl electrodes arranged in equidistant positions (1000 Hz sampling rate, ground electrode at *θ* = 58, *φ* = 78, reference electrode at Fpz) using the BrainVision Recorder software. All electrode impedances were kept below 5 kΩ. The BrainVision Analyzer II was used for data pre-processing and analysis. Offline, the EEG was down-sampled to 256 Hz. Data were manually inspected to remove technical artifacts. A band-pass filter (0.5–20 Hz at 48 db/oct each) was applied. To correct for periodically recurring artifacts (horizontal and vertical eye movements and blinks), an independent component analysis (ICA; infomax algorithm) was applied to the unepoched data sets. Components that revealed horizontal and vertical eye movements and blinks were visually identified by means of recurrent similar waveform and by means of scalp topography. ICA components reflecting the above-mentioned artifacts were then discarded. The EEG was segmented into compatible and incompatible trials under the constraint that the response given in the trials was correct. Trial segmentation was target-locked and began 2000 ms before the target presentation of the respective trial and ended 2000 ms after its presentation. Afterwards, an automated artifact rejection procedure using a maximum/minimum amplitude of 100/−100 μV as well as an activity below 0.5 μV in a 200 ms period as rejection criteria was applied. A current source density (CSD) transformation (Nunez and Pilgreen, 1991) was utilized to eliminate the reference potential from the data. For the baseline correction, a 200 ms time interval before target stimulus presentation was utilized. At this point it is important to note that another baseline (i.e., from −400 ms until −200 ms; i.e., before flanker stimuli presentation) did not change the pattern of results in the neurophysiological data. Averages were calculated separately for all conditions at the single subject level. Data quantification was conducted on the single subject data. Specific electrodes and time windows were selected on the basis of the scalp topography using a data-driven approach. The classic ERPs were examined in corresponding time windows. The P1_Flanker_ (−95 ms to −75 ms), the N1_Flanker_ (−10 ms to 10 ms), the P1_Target_ (115–135 ms) and the N1_Target_ (260–280 ms) were quantified at electrodes P9 and P10. The N2 was measured at 280–320 ms over electrodes FCz and Cz and the P3 was examined at electrode Pz at 300–320 ms. This choice was validated using a statistical approach outlined in Mückschel et al. (2014) for each group separately (Chmielewski et al., 2014). Doing so, the above time intervals were taken and the mean amplitude within the defined search intervals was determined for each of the 60 electrode positions. Then, to compare activation recorded at each electrode against the average of activation recorded at all other electrodes, Bonferroni correction for multiple comparisons (critical threshold, *p* = 0.0007) was used. Only electrodes at which recorded activation was significantly stronger (i.e., negative for the N-potentials and positive for the P-potentials) when compared to that at other electrodes were chosen. This procedure revealed the same electrodes of interest as those chosen by visual inspection.

The RIDE decomposition was performed according to established procedures (Ouyang et al., 2011; Verleger et al., 2014) using the RIDE toolbox and manual available on http://cns.hkbu.edu.hk/RIDE.htm. RIDE decomposes EEG data on the basis of their timing and timing variability properties. Because RIDE only makes use of latency variability and separates component clusters irrespective of their scalp distributions and waveforms (Ouyang et al., 2015a,b), the application of the CSDs that work as a spatial filter is not critical. The time markers (“latencies”) used for deriving the S and R cluster components (“LS” and “LR”) are the time points of the stimulus and response onsets, respectively. In contrast to this, the time markers for deriving C (“LC”) are estimated and iteratively improved. RIDE uses a time window function to extract the waveform of each RIDE component. For the current study this was from 200 ms prior to target to 600 ms after the target for the S-cluster, from 200 ms to 900 ms after the target for the C-cluster and ±300 ms around the response trigger for the R-cluster (Ouyang et al., 2015a). To estimate S, RIDE subtracts C and R from each single trial and aligns the residual of all trials to the latency “LS” in order to obtain S as the median waveform for all time points. The equivalent procedure is applied to obtain C and R. For the R-cluster the response needs to be part of the epoch and around 98% of all responses were carried out within the epoch. The whole procedure is iterated to improve the estimation of the components until they converge (criterion: less than 10^−3^ difference for the values of two successive iterations). Full details on the RIDE method can be found in Ouyang et al. (2011, 2015a).

The three RIDE clusters can be used to quantify components corresponding to classic ERPs. Just like it was the case for the traditional ERP analysis, we also applied a data-driven approach to quantify appropriate time windows and topographic locations for the RIDE-components (2014). In our case, the R-cluster was examined at electrodes C3 and C4 in the time window of ±10 ms around the average reaction times (RTs) in the compatible and incompatible trials. In the S-cluster, we quantified the RIDE-P1_Flanker_ (−95 ms to −75 ms), the RIDE-N1_Flanker_ (−10 ms to 10 ms), the RIDE-P1_Target_ (115–135 ms) and the RIDE-N1_Target_ (260–280 ms) at electrodes P9 and P10. In addition, the S-cluster RIDE-N2 (N2_S_) was examined over electrodes FCz and Cz at 280–320 ms. As reported previously (Ouyang et al., 2011, 2015a,b; Verleger et al., 2014), the latencies and topographies of the ERP- and the RIDE-components were identical in the S-cluster, as the close proximity to the common locking point leads to a relatively low impact of the intra-individual variability. In the C-cluster, the RIDE-N2 (N2_C_) was quantified in the time window of 370–390 ms over electrodes FCz and Fz. The C-cluster RIDE-P3 was measured over electrode Pz. Time windows (ADD: 520–540 ms; ADHD-C: 590–610 ms) were adapted based on the observed peak latencies. As it was to be expected (Ouyang et al., 2011, 2015a,b; Verleger et al., 2014), the component characteristics in the RIDE analysis were different to those in the ERP analysis. This is due to the larger impact of the variability, which arises due to the larger time interval to the common locking point. The choice of time windows and electrodes quantified for the RIDE decomposition was also validated using the statistical approach outlined in Mückschel et al. (2014).

### Source Localization Analysis

For the source localization analysis, standardized low resolution brain electromagnetic tomography (sLORETA; Pascual-Marqui, 2002) was used. As a basis for the source localization analysis we used the estimated RIDE cluster. sLORETA provides a single solution to the inverse problem (Pascual-Marqui, 2002; Marco-Pallarés et al., 2005; Sekihara et al., 2005) based on the MNI152 template (Mazziotta et al., 2001). It has been mathematically proven that sLORETA provides reliable results without a localization bias (Sekihara et al., 2005) and also combined TMS/EEG studies support this (Sekihara et al., 2005; Dippel and Beste, 2015). The voxel-based sLORETA images were compared across compatibility conditions and groups using the sLORETA-built-in voxel-wise randomization tests with 2000 permutations, based on statistical nonparametric mapping (SnPM). Voxels with significant differences (*p* < 0.01, corrected for multiple comparisons) between contrasted conditions were located in the MNI-brain^1^.

### Statistics

Descriptive statistics are shown as mean ± standard error. The data was analyzed using mixed effects analysis of variance (ANOVAs) using *Group* (ADD vs. ADHD-C) as the between-subject factor. As a within-subject factor, *Compatibility* (compatible vs. incompatible trials) was modeled to account for the two different flanker-target combinations (not applicable to (RIDE-)P1_Flanker_ and (RIDE-)N1_Flanker_). Where necessary, *Electrode* was modeled as an additional within-subject factor and is only reported when a relevant interaction with *Group* was observed. Greenhouse-Geisser correction was applied for all ANOVAs. *Post hoc* tests were Bonferroni-corrected when necessary. All variables included in the analysis were normally distributed, as indicated by Kolmogorov-Smirnov tests (all *z* < 1.05; *p* > 0.3).

## Results

### Behavioral Data

As groups were compiled specifically to not display any differences on the behavioral level, there was only a main effect of *Compatibility* (*F*_(1,57)_ = 65.9; *p* ≤ 0.001; $\eta_{\text{p}}^{\text{2}}$ = 0.54) in RTs, showing faster responses in compatible (339 ± 10 ms) compared to incompatible trials (402 ± 15 ms; all other *F* ≤ 0.48; *p* ≥ 0.49; $\eta_{\text{p}}^{\text{2}}$ = 0.008). Similar results were found for accuracy. The main effect of *Compatibility* was significant (*F*_(1,57)_ = 64.6; *p* ≤ 0.001; $\eta_{\text{p}}^{\text{2}}$ = 0.53), with more correct responses in compatible (66.2 ± 3.0%) than in incompatible trials (39.7 ± 2.5%; all others *F* ≤ 0.5; *p* ≥ 0.48; $\eta_{\text{p}}^{\text{2}}$ = 0.009). Moreover, further analyses show that performance on incompatible trials was significantly below chance level in each of the investigated groups (*p* < 0.001). Regarding intra-individual variability, we calculated the within-subject standard deviation of the RTs at the single subject level and analyzed this data using mixed ANOVAs. The results show only a main effect *Compatibility* (*F*_(1,57)_ = 5.86; *p* = 0.019; $\eta_{\text{p}}^{\text{2}}$ = 0.093) with the variability being higher on incompatible (134 ± 4.7 ms) than compatible trials (124 ± 6.5 ms). There were no main effects or interactions involving the factor *Group* (all *F* ≤ 0.008; *p* ≥ 0.9).

### Neurophysiological Data

#### Event-Related Potentials (ERPs)

**Figure 2:**
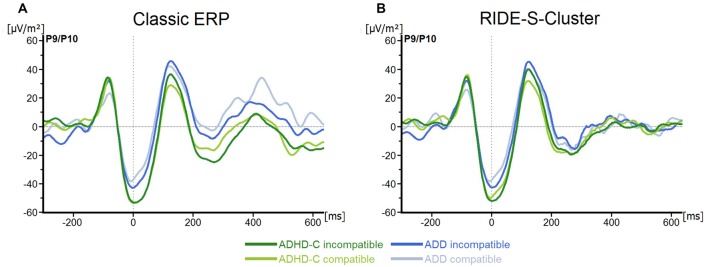
**(A)** Stimulus-locked waveforms (current source density, CSD) for P1_Flanker_, N1_Flanker_, P1_Target_, N1_Target_ components and **(B)** the equivalent residue iteration decomposition (RIDE)-components (S-Cluster), depicted for both attention deficit/hyperactivity disorder (ADHD) subtypes and for compatible and incompatible flanker trials at electrodes P9/P10. Point 0 denotes the onset of the target stimulus.

No main effects or interactions were statistically significant for the P1_Flanker_ (all *F* ≤ 1.0; all *p* ≥ 0.32; all $\eta_{\text{p}}^{\text{2}}$ ≤ 0.02), the N1_Flanker_ (all *F* ≤ 2.9; all *p* ≥ 0.1; all $\eta_{\text{p}}^{\text{2}}$ ≤ 0.05), the P1_Target_ (all *F* ≤ 2.1; all *p* ≥ 0.15; all $\eta_{\text{p}}^{\text{2}}$ ≤ 0.04) or the N1_Target_ (all *F* ≤ 3.5; all *p* ≥ 0.07; all $\eta_{\text{p}}^{\text{2}}$ ≤ 0.06; see Figure 2A).

**Figure 3:**
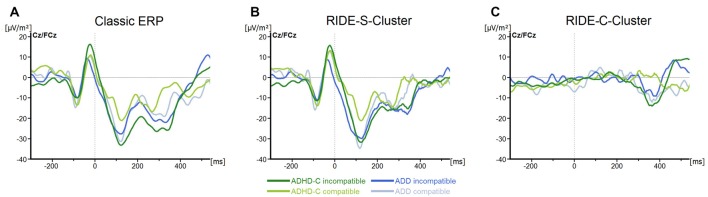
**(A)** Stimulus-locked waveforms (CSD) for the N2 component and **(B)** the equivalent RIDE-components in the S-Cluster) and **(C)** the C-Cluster, depicted for both ADHD subtypes and for compatible and incompatible flanker trials at electrodes Cz/FCz. Point 0 denotes the onset of the target stimulus.

For the N2, we only found a main effect of *Compatibility (F*_(1, 57)_ = 6.8; *p* = 0.01; $\eta_{\text{p}}^{\text{2}}$ = 0.1), with the component being more pronounced in incompatible (−23.4 ± 3.7 μV/m^2^) than in compatible trials (−14.1 ± 3.6 μV/m^2^; all others *F* ≤ 3.1; all *p* ≥ 0.09; all $\eta_{\text{p}}^{\text{2}}$ ≤ 0.05; see Figure 3A).

**Figure 4:**
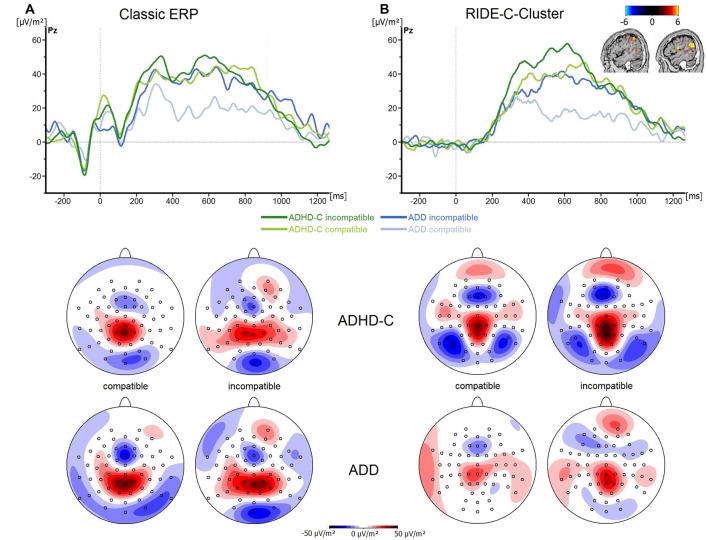
**(A)** Stimulus-locked waveforms (CSD) for the P3 component and **(B)** the equivalent RIDE-component, depicted for both ADHD subtypes and for compatible and incompatible flanker trials at electrode Pz. Point 0 denotes the onset of the target stimulus. Topographic maps are also shown—blue denotes negative deflections whereas red reflects positive ones.

Finally, the analysis of the P3 revealed no significant main effects or interactions (all *F* ≤ 3.3; all *p* ≥ 0.07; all $\eta_{\text{p}}^{\text{2}}$ ≤ 0.06; see Figure 4A). Even though the choice of the electrode sites and time windows was validated using statistical methods, the time windows for data quantification in the P3 ERP differed from those used in the RIDE C-cluster (see “Materials and Methods” Section). To rule out that the pattern of results changed when the same time windows were used for data analysis, we analyzed the P3 ERP data in the same time windows as used for the RIDE C-cluster data. This analysis revealed no main effects or interactions (*F* ≤ 1.4; all *p* ≥ 0.34; all $\eta_{\text{p}}^{\text{2}}$ ≤ 0.03). This shows that the effects obtained in the RIDE C-cluster data (see below) are not due to a bias related to different time intervals used for data quantification.

#### Residue Iteration Decomposition (RIDE)

**Figure 5:**
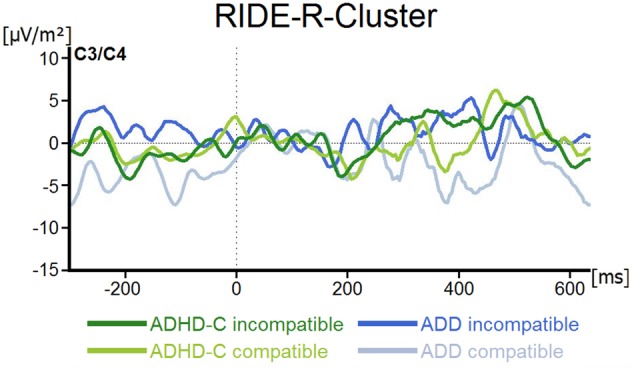
R-Cluster RIDE-component depicted for both ADHD subtypes and for compatible and incompatible flanker trials at electrodes C3/C4. Point 0 denotes the onset of the target stimulus.

For the R-component measured at electrodes C3 and C4, the mixed effects ANOVA revealed no main or interaction effects (all *F* ≤ 2.5; all *p* ≥ 0.12; all $\eta_{\text{p}}^{\text{2}}$ ≤ 0.04; see Figure 5).

For the S-cluster, the repeated measures ANOVA revealed no effects of any of the factors on RIDE-P1_Flanker_ (all *F* ≤ 2.6; all *p* ≥ 0.11; all $\eta_{\text{p}}^{\text{2}}$ ≤ 0.043), RIDE-N1_Flanker_ (all *F* ≤ 3.3; all *p* ≥ 0.07; all $\eta_{\text{p}}^{\text{2}}$ ≤ 0.06), RIDE-P1_Target_, (all *F* ≤ 2.5; all *p* ≥ 0.1; all $\eta_{\text{p}}^{\text{2}}$ ≤ 0.042) or RIDE-N1_Target_, (all *F* ≤ 1.4; all *p* ≥ 0.24; all $\eta_{\text{p}}^{\text{2}}$ ≤ 0.03; see Figure 2B). Concerning the S-cluster N2 (Figure 3B), the mixed effects ANOVA only showed an interaction of Group*Electrode (*F*_(1,57)_ = 4.8; *p* = 0.03; $\eta_{\text{p}}^{\text{2}}$ = 0.08), but further *post hoc* testing did not withstand Bonferroni-corrections (*p* > 0.2; all others *F* ≤ 2.5; all *p* ≥ 0.12; all $\eta_{\text{p}}^{\text{2}}$ ≤ 0.04). Thus, the results of the RIDE analysis very closely reflect those of the traditional ERP analysis. The reason for this is the close proximity to the locking point (stimulus), which results in the variability having only a small effect.

Regarding the C-cluster RIDE-N2 (see Figure 3C), no significant main effects or interactions were evident (all *F* ≤ 3.8; all *p* ≥ 0.06; all $\eta_{\text{p}}^{\text{2}}$ ≤ 0.06). Opposed to this, the analysis of the C-cluster RIDE-P3 (see Figure 4B) revealed a main effect of *Compatibility* (*F*_(1,57)_ = 34.6; *p* ≤ 0.001; $\eta_{\text{p}}^{\text{2}}$ = 0.38) with the RIDE-P3 being larger for incompatible (48.2 ± 4.5 μV/m^2^) than for compatible trials (26.5 ± 4.4 μV/m^2^). Furthermore, there was a significant main effect of *Group* (*F*_(1,57)_ = 6.4; *p* = 0.014; $\eta_{\text{p}}^{\text{2}}$ = 0.1), showing that the ADHD-C group generally had a larger RIDE-P3 (47.5 ± 5.2 μV/m^2^) than the ADD group (27.2 ± 6.1 μV/m^2^). Interestingly, there was also a significant interaction of Compatibility*Group (*F*_(1,57)_ = 4.9; *p* = 0.03; $\eta_{\text{p}}^{\text{2}}$ = 0.08). *Post hoc* tests showed that the compatibility (conflict) effect was larger (29.7 ± 6.7 μV/m^2^) in the ADD group than in ADHD-C group (13.5 ± 3.9 μV/m^2^; *t*_(57)_ = 2.2; *p* = 0.016). This effect was mainly driven by the compatible trials. There, the patients with ADD group showed a smaller RIDE-P3 (12.2 ± 7.1) than those with ADHD-C (40.7 ± 5.2; *t*_(57)_ = −3.23; *p* = 0.001). There was no group difference on incompatible trials (*t*_(57)_ = −1.36; *p* = 0.18; ADD: 42.0 ± 6.2; ADHD-C 54.3 ± 6.2). Correlational analyses revealed no connection between RIDE-P3 and severity of ADHD symptoms (all −0.08 ≤ *r* ≤ 0.19, all *p* ≥ 0.14). The sLORETA analysis shows that the modulation in the conflict effect was related to activity modulations in the inferior parietal cortex and the temporo-parietal junction (TPJ; BA40) in particular. Thus, the results of the RIDE analysis differ significantly from those of the traditional ERP analysis.

To control whether the specificity of effects obtained for the C-cluster data is an effect of a systematic bias in the signal-to-noise ratio (SNR) attributable to one specific group, we calculated the SNR for each condition, electrode and RIDE-cluster. We calculate the SNR as implemented in the Brain Vision Analyzer II as previously done in other studies (Beste et al., 2015; Gohil et al., 2016). The results show that in all analyzed RIDE-clusters and electrodes, there was only a main effect of *Compatibility* (all *F* > 11.41; *p* < 0.001; $\eta_{\text{p}}^{\text{2}}$ = 0.251) showing that the SNR was higher in compatible (1.15 ± 0.5) than incompatible trials (1.01 ± 0.3). This is a trivial finding because the SNR is affected by the number of trials included in the analysis. Importantly, there were no other main effects or interactions (all *F* < 1.05; *p* > 0.32). This shows that the SNR does not pose a systematic bias in the data analysis regarding any main effects or interactions with the factor *Group*. This underlines the reliability of the obtained results.

## Discussion

We examined differences in the intra-individual variability of cognitive-neurophysiological processes related to conflict monitoring between patients with ADD and ADHD-C. Importantly, we used RIDE (Ouyang et al., 2011, 2015a,b; Verleger et al., 2014) to examine differences in neural activity independent of the strong intra-individual variability that may bias insights into such processes. This is important because of the inherent high intra-individual variability in ADHD (Henríquez-Henríquez et al., 2014; Lin et al., 2015; Saville et al., 2015). To further control for this variability factor, we specifically compared patients with ADD and ADHD-C that did not differ in behavioral performance.

There were no differential modulations between groups and conditions in neurophysiological processes related to the sensory and attentional processing of stimuli and to primary motor processes. Thus the characteristic differences between the two groups on the symptom level (specifically concerning hyperactivity/impulsivity) are not connected to differences in basic sensory or attentional processing. This was the case independent of the applied methodology (standard ERPs vs. RIDE-ERPs), showing that this finding does not seem to be influenced by the characteristic high intra-subject variability. Also, due to the close proximity to the common locking point, the effects of variability are generally rather small in this time window (Ouyang et al., 2011, 2015a,b; Verleger et al., 2014; Mückschel et al., 2017). This is equally the case for primary motor processes. Here, the RIDE analysis also did not reveal any differences between the two ADHD subgroups, thus supporting findings suggesting that any problems in motor execution in ADHD are independent of the subtype (Ghanizadeh, 2010).

In contrast, the RIDE analysis (C-cluster) did indeed show significant differences between subgroups in conflict-related neurophysiological processes. This effect was not biased by issues relating to differences in SNRs. The fact that these results did not become apparent in the standard ERP analysis very likely reflects the strong intra-individual variability inherent to ADHD (Henríquez-Henríquez et al., 2014; Lin et al., 2015; Saville et al., 2015). Importantly, also from a conceptual/methodological point of view, the finding that only the RIDE-P3 shows differences between groups makes much sense. It has been shown that intra-individual variability mostly affects long latency ERP components (Ouyang et al., 2011, 2015a,b; Verleger et al., 2014). So, if the intra-individual variability is different between groups, it is to be expected that particularly the P3 shows differences. The obtained specific group effect found in this study (which was specific to the P3) is what has to be expected. This conceptual aspect makes it very unlikely that the result reflect a false positive effect and also recent analyses suggest that especially findings concerning the P3 are influenced by RIDE analyses, independent of the paradigm applied (Ouyang et al., 2017; Wolff et al., 2017).

When neuronal variability was accounted for, differences between ADHD subtypes actually did emerge. In this regard, the source localization analysis shows that differences between ADD and ADHD-C subtypes are associated with sources localized in the inferior parietal cortex (BA40) including the TPJ. The parietal cortex has been shown to reveal structural and functional abnormalities in ADHD (Bush et al., 2013). The TPJ has also been shown to be associated with the P3 ERP in the ADHD patients enrolled in this study (refer source localization results), which however did not show differences between subtypes. These results suggest that there is a strong variability of neuronal processes in ADHD that relates to the inferior parietal cortex and the TPJ. Interestingly, the C-cluster has been shown to reflect processes that are functionally similar to those attributed to the classic P3 ERP (Verleger et al., 2014). The P3 is well-known to reflect decision processes occurring between stimulus- and response-related mechanisms, (Mückschel et al., 2014; Verleger et al., 2014), for which the TPJ plays an important role (Geng and Vossel, 2013; Mückschel et al., 2014; Verleger et al., 2014). It has also been suggested that the P3—and thus possibly also the C-cluster—reflects mechanisms of accumulating evidence needed for reaching a decision about the response to be selected (Twomey et al., 2015). The present results suggest that this process takes place in two different manners in the examined patient groups and that thus, differences between the groups may not be as straightforward as initially assumed. ADD patients seem to be able to achieve a distinction between compatible and incompatible trials more efficiently than ADHD-C patients. This is supported by the fact that the difference in C-cluster amplitudes in compatible trials only was twice as large in ADD as in ADHD-C. When considering the latency of the C-cluster it becomes apparent that this process of distinguishing between compatible and incompatible trials occurs relatively early on in patients with ADD. In patients with ADHD-C, no such distinction between compatible and incompatible trials is taking place, suggesting that evidence accumulation functions differently in ADHD-C. Patients with ADHD-C generally had stronger C-Cluster activations, possibly pointing towards more cognitive resources being invested during decision-making. This could be interpreted as a more automated response selection strategy that functions efficiently even without a clear distinction between compatible and incompatible trials. Thus, the two examined experimental groups reach the same behavioral outcome through two different manners of accumulating the evidence needed to reach a decision for a response. These results thus render further support for the idea that patients with ADD and ADHD-C may not just simply differ in their behavior, but actually are characterized by different underlying information processing strategies. These may not be apparent at first glance, but may still carry important implications for clinical practice. For example, knowledge of such differences could, in the future, be applied to achieve improvements of the diagnostic process. Applying methods that quantify and account for intra-individual variability in neurophysiological data may also prove useful in clinical trials trying to establish neurophysiological biomarkers of ADHD subtypes.

Of course, this study also is not without limitations. First, no correlations were evident between the neurophysiological and clinical measures. In this regard it needs to be noted that the reliability of the different correlated measures is an issue that needs to be considered. However, the current study shows that some differences between ADD and ADHD-C subtypes might not be reflected in routinely used clinical measures and therefore may remain unnoticed. Second, it may be argued that parts of the above interpretation concerning the nature of processes differentially modulated in ADD and ADHD-C are based on reverse inference (Poldrack, 2006, 2011), as no behavioral effects were evident. However, the functional relevance of the examined neurophysiological correlates has been well-established in the applied task (van Meel et al., 2007; Albrecht et al., 2008; Mullane et al., 2009; Beste et al., 2010; Chmielewski et al., 2014). Third, error rates in the current task were rather high. However, the reported main effect of *Condition* clearly shows that patients were not just responding randomly (as they could distinguish between compatible and incompatible trials). The low accuracy rates overall can thus not simply be attributed to the ADD/ADHD-C diagnoses. Lastly, no patients with the predominantly hyperactive/impulsive ADHD subtype were included in the study. Doing so would allow a better differentiation of the effects of inattentiveness and hyperactivity/impulsivity on executive functions generally and on conflict processing in particular.

In summary, the study shows that there are differences in neuronal mechanisms related to response selection processes between ADD and ADHD-C. Importantly, these differences in neuronal processes were only detected when methodologically accounting for intra-individual variability. We were thus able to show that differences between the examined ADHD subtypes not only occur on the symptom level, but are also clearly reflected on the neuronal level. These findings illustrate the need to changes perspectives, to look beyond pure behavioral performance and to take alterations in intra-individual variability into account when interpreting data. An unbiased consideration of underlying neuronal processes adds crucial information concerning inter-individual differences within one diagnostic category, just like suggested by the RDoC initiative (Insel et al., 2010). The results imply that it is very likely that differences in neurophysiological processes between ADHD subtypes are underestimated and have not been recognized because variability in neurophysiological data has not sufficiently been taken into account. It is therefore important that methods being able to control intra-individual variability in neurophysiological data are increasingly considered to avoid biases in knowledge on neurophysiological processes in ADHD. The same may apply to existing data.

## Author Contributions

AB and CB conceived and designed the study, analyzed the data and wrote the article. AB, MM and WXC analyzed data and wrote the article. VR conceived the study and wrote the article.

## Conflict of Interest Statement

The authors declare that the research was conducted in the absence of any commercial or financial relationships that could be construed as a potential conflict of interest.

## Footnotes

^1^www.unizh.ch/keyinst/NewLORETA/sLORETA/sLORETA.htm

## Abbreviations

ADD: attention deficit disorder
ADHD-C: attention deficit/hyperactivity disorder—combined subtype
ERP: event-related potentials
RIDE: residue iteration decomposition.
